# A Patient Decision Aid for Anticoagulation Therapy in Patients With Nonvalvular Atrial Fibrillation: Development and Pilot Study

**DOI:** 10.2196/23464

**Published:** 2021-08-12

**Authors:** Kim Paul de Castro, Harold Henrison Chiu, Ronna Cheska De Leon-Yao, Lorraine Almelor-Sembrana, Antonio Miguel Dans

**Affiliations:** 1 Department of Medicine Philippine General Hospital University of the Philippines Manila Philippines; 2 Department of Adult Cardiology Philippine Heart Center Quezon City Philippines; 3 Division of Adult Medicine, Department of Medicine Philippine General Hospital University of the Philippines Manila Philippines

**Keywords:** shared decision-making, patient decision aid, atrial fibrillation, anticoagulation, stroke prevention, mHealth, mobile health

## Abstract

**Background:**

Atrial fibrillation (AF) is one of the most common predisposing factors for ischemic stroke worldwide. Because of this, patients with AF are prescribed anticoagulant medications to decrease the risk. The availability of different options for oral anticoagulation makes it difficult for some patients to decide a preferred choice of medication. Clinical guidelines often recommend enhancing the decision-making process of patients by increasing their involvement in health decisions. In particular, the use of patient decision aids (PDAs) in patients with AF was associated with increased knowledge and increased likelihood of making a choice. However, the majority of available PDAs are from Western countries.

**Objective:**

We aimed to develop and pilot test a PDA to help patients with nonvalvular AF choose an oral anticoagulant for stroke prevention in the local setting. Outcomes were (1) reduction in patient decisional conflict, (2) improvement in patient knowledge, and (3) patient and physician acceptability.

**Methods:**

We followed the International Patient Decision Aid Standards (IPDAS) to develop a mobile app–based PDA for anticoagulation therapy in patients with nonvalvular AF. Focus group discussions identified decisional needs, which were subsequently incorporated into the PDA to compare choices for anticoagulation. Based on recommendations, the prototype PDA was rendered by at least 30 patients and 30 physicians. Decisional conflict and patient knowledge were tested before and after the PDA was implemented. Patient acceptability and physician acceptability were measured after each encounter.

**Results:**

Anticoagulant options were compared by the PDA using three factors that were identified (impact on stroke and bleeding risk, and price). The comparisons were presented as tables and graphs. The prototype PDA was rendered by 30 doctors and 37 patients for pilot testing. The mean duration of the encounters was 15 minutes. The decisional conflict score reduced by 35 points (100-point scale; *P*<.001). The AF knowledge score improved from 10 to 15 (*P*<.001). The PDA was acceptable for both patients and doctors.

**Conclusions:**

Our study showed that an app-based PDA for anticoagulation therapy in patients with nonvalvular AF (1) reduced patient decisional conflict, (2) improved patient knowledge, and (3) was acceptable to patients and physicians. A PDA is potentially acceptable and useful in our setting. A randomized controlled trial is warranted to test its effectiveness compared to usual care. PDAs for other conditions should also be developed.

## Introduction

Atrial fibrillation (AF) is the most common sustained cardiac dysrhythmia [1] and has been recognized as one of the most common causes of ischemic stroke. In a global survey published in 2016, it was found that the frequency of AF-associated stroke was 28% [2]. The risk of developing a stroke from AF can be decreased with the use of anticoagulation therapy. However, almost half of all patients with AF were shown to never start or continue their anticoagulant medications to prevent the occurrence of a stroke [3-5]. Currently, multiple drug options with different efficacy and safety profiles exist in the market. Therefore, it is imperative for health care providers to consider their patients’ individual preferences and involve them in the decision-making process.

Shared decision-making (SDM) has been promoted as a helpful adjunct in health care delivery, especially when multiple treatment options with varied outcomes exist. This point in the decision-making process creates a state of uncertainty known as decisional conflict [6]. In order to facilitate SDM, patient decision aids (PDAs) that take into consideration the patient’s values and needs have been advocated to encourage a two-way exchange of information between the health care provider and the patient [7-9]. Among patients with AF, the use of PDAs was associated with increased patient knowledge, increased likelihood of making a choice, and low decisional conflict [10]. This is particularly important because patients whose values and preferences were not taken into consideration in the decision-making process were shown to be more dissatisfied and nonadherent to therapy, hence increasing the risk for ischemic stroke [4,11,12]. The latest cardiovascular clinical guidelines already recommend the use of SDM to facilitate an individualized approach to anticoagulation therapy in patients with nonvalvular AF [13].

While the benefits of using PDAs have been validated in Western countries, SDM still remains a novel concept in Asia where paternalism is still the norm [14]. Nevertheless, it has been found that patients in Asia want to be involved in SDM [15,16]. In the Philippines, there has only been one published study on a PDA that was developed for diabetic patients choosing an oral hypoglycemic agent [17]. This study found that PDAs are feasible to use in a lower middle–income country without significantly increasing consult time. To date, no PDA for anticoagulation therapy in AF has been developed and published in our setting. We developed a PDA to address this gap.

Specifically, we aimed to develop and pilot test a mobile app–based decision aid that focuses on supporting the decision-making process of patients with nonvalvular AF regarding their choice of oral anticoagulation therapy in our setting. As a pilot test, our study sought to determine the level of decisional conflict and knowledge among patients at baseline and after administration of the decision aid, and to assess the acceptability of our decision aid among doctors and patients in the local setting.

## Methods

### Overview

**Figure 1:**
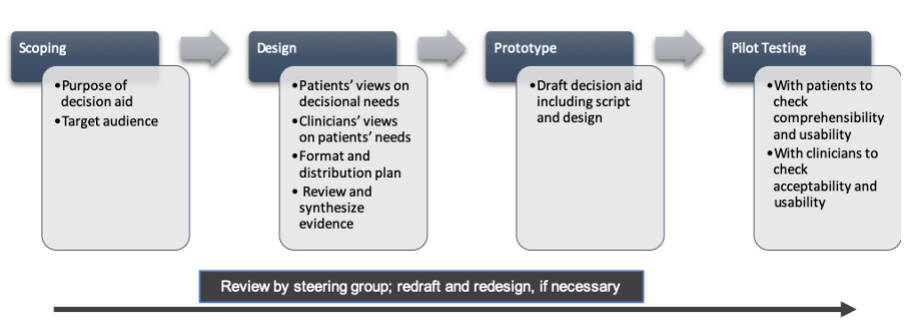
Decision aid development process.

This study encompasses the first two parts of the PDA development process, as outlined by The International Patient Decision Aid Standards (IPDAS) [8]. It was executed in two phases. Phase I involved literature review and focus group discussions (FGDs) among patients and doctors, which assessed their values and decisional needs. The product of phase I was a prototype application-based PDA that was used in the implementation of phase II. In phase II, pilot testing was done to assess the PDA’s acceptability for doctors and patients and its effect on patients’ knowledge and decisional conflicts. This pilot testing employed a before-and-after study with the aid of validated outcome assessment tools from the Ottawa Decision Support Framework [6,18,19]. The University of the Philippines – Philippine General Hospital (UP-PGH) Expanded Hospital Research Office approved this study. Figure 1 shows the general flow of the development process.

### Phase I: Creation of a PDA Prototype

#### Decisional Needs Assessment

Patients were selected through convenience sampling from the UP-PGH General Medicine, Cardiology, and Neurology Outpatient Clinics. Recruitment was done in the following two ways: (1) through physicians from the abovementioned outpatient clinics who identified patients with nonvalvular AF, and (2) research assistants who were stationed outside the said clinics who actively asked patients if they have “irregular beating of the heart” or are taking anticoagulants. Once assessed by the investigators as qualified, a trained research assistant explained the informed consent, and those who consented were included in the FGDs.

Physicians who were included for the needs assessment were doctors from different specialties who were direct health care providers for patients with AF. They were mainly recruited from the residents and fellows in training under Internal Medicine, Cardiology, and Neurology.

FGDs were conducted with both patients and physicians to identify the decision-making issues when dealing with patients with AF. The discussions were centered on the overall theme of SDM, its applicability to the local setting, and factors that patients and doctors consider important when faced with the different options of anticoagulation for stroke prevention.

Two FGDs with six to eight participants were conducted to identify the decisional needs. After informed consent was obtained from each participant, a moderator facilitated the discussion using a set of guide questions. Both sessions were recorded and transcribed verbatim by a research assistant, and the data were processed for analysis.

#### Drafting of the PDA Prototype

The authors served as the steering group of clinicians who guided the drafting of the PDA prototype in all its stages of development. Information gathered from the aforementioned FGDs that identified decisional needs were incorporated in the prototype PDA. A systematic literature search was done for evidence on the effectiveness, bleeding risk, adverse effects, and dosing of the different medications available in the Philippines. The available literature that was used in the study was appraised for directness, validity, and applicability prior to inclusion into the evidence base of the PDA [20-22]. The cost of the included medications was surveyed from the largest chain of pharmacies in the country. A modified version of a validated image [23] for patient education was also incorporated into the PDA prototype. These plans were communicated through a series of meetings with an independent third-party contractor that was commissioned to program the PDA into a mobile-based app.

### Phase II: Pilot Testing

A prospective before-and-after observational design was used to pilot test the PDA. Doctors and patients from the UP-PGH General Internal Medicine, Neurology, Cardiology, and Faculty Clinics were invited to participate in pilot testing through convenience sampling. Eligible patients included adults who were at least 19 years old, were able to speak and understand Filipino and English, and had a diagnosis of nonvalvular AF. Patients with major cognitive or psychiatric symptoms were excluded. Similar to phase I, recruitment was done in the following two ways: (1) through physicians who referred their patients with nonvalvular AF, and (2) through research assistants who were stationed outside the said clinics who actively asked patients if they have “irregular beating of the heart” or are taking anticoagulants. Once deemed eligible, written informed consent was obtained prior to enrollment. A convenience sample of 30 doctors and 37 patients participated. Prior to the actual patient-doctor encounter, a special training session was held to give the physicians a trial copy of the PDA, introduce them to the interface, and allow them to practice using the mobile app. On the day of the patient-doctor encounter, the physicians were instructed to use the PDA to facilitate patient education and decision-making regarding anticoagulation therapy.

Before the encounter, data collected from patients included demographics, baseline knowledge about AF using the Ottawa PDA Knowledge Tool, and baseline level of decisional conflict measured using the Ottawa PDA Decisional Conflict Scale (DCS). After the encounter, patients were again asked to answer the Ottawa Knowledge Tool and Ottawa PDA DCS [6,18]. Both patients and doctors were also asked to answer the Ottawa Acceptability Tool–Patient and Practitioner Versions [19]. These validated outcome assessment tools were administered via an interviewer-assisted questionnaire. In order to protect the participants’ information, names were anonymized and the data collection forms were coded and kept in a locked cabinet.

The outcomes for this pilot study were the developed PDA’s effects on patient knowledge, the level of decisional conflict, and patient and physician acceptability. Descriptive statistics using means and SDs were used for continuous variables, and proportions were used for categorical variables. Bivariate analysis included paired *t* tests for comparing pre-PDA and post-PDA knowledge and decisional conflict scores.

## Results

### Decisional Needs Assessment: Patients

A FGD was conducted wherein eight patients (five female and three male patients) participated. They were all diagnosed with nonvalvular AF and were taking anticoagulant medications (five were on warfarin and three were on rivaroxaban). Important points that emerged during the discussion were as follows:

1. Participants knew that AF is the irregular beating of the heart, but they were unsure what causes the condition.
2. The bad outcomes of AF that patients knew were ischemia (ie, stroke and transient ischemic attacks) and symptoms of heart failure (ie, easy fatigability, dyspnea, and orthopnea). Awareness of these conditions causes anxiety.
3. The prevention of bad outcomes entails intake of anticoagulants and regular follow-up with health care providers.
4. Factors taken into consideration when choosing anticoagulant medications are efficacy, side effects (ie, risk of bleeding and gastrointestinal upset), cost, frequency of laboratory testing, and doctor’s preference.
5. Benefits associated with the intake of drugs include anticoagulation, stroke prevention, and better sense of well-being (ie, tolerate exercise). Unwanted consequences of taking medications include bleeding, frequent laboratory testing, and diet modifications.
6. The participants had different opinions on SDM. A majority would like to be involved in health care decisions, while some still subscribed to the paternalistic doctor-patient relationship.

### Decisional Needs Assessment: Doctors

Six physicians (three internists, two neurologists, and one cardiologist) participated. Salient points that surfaced during the discussion were as follows:

1. The doctor-patient relationship is a partnership. Providing patients with enough knowledge is important for them to make an informed choice when it comes to their health. Once a decision has been made, patient autonomy should always be respected.
2. Patients are uncomfortable with initiating anticoagulants due to frequent blood tests, adverse effects of the drugs, and associated costs. A better understanding of AF and the need for anticoagulation makes it easier to convince them to start anticoagulants.
3. In helping patients choose their medications, it is crucial to tell them about the risks, benefits, frequency of laboratory tests, ease of compliance, and cost of the drugs. The availability of a reversal agent should also be mentioned.
4. Support from family and the health care provider helps patients make decisions regarding their health.
5. Different strategies, such as using visual aids, modeling, presenting real-world data, and enlisting the help of patient education advocates, can help patients in choosing an anticoagulant.

### PDA Prototype

**Figure 2:**
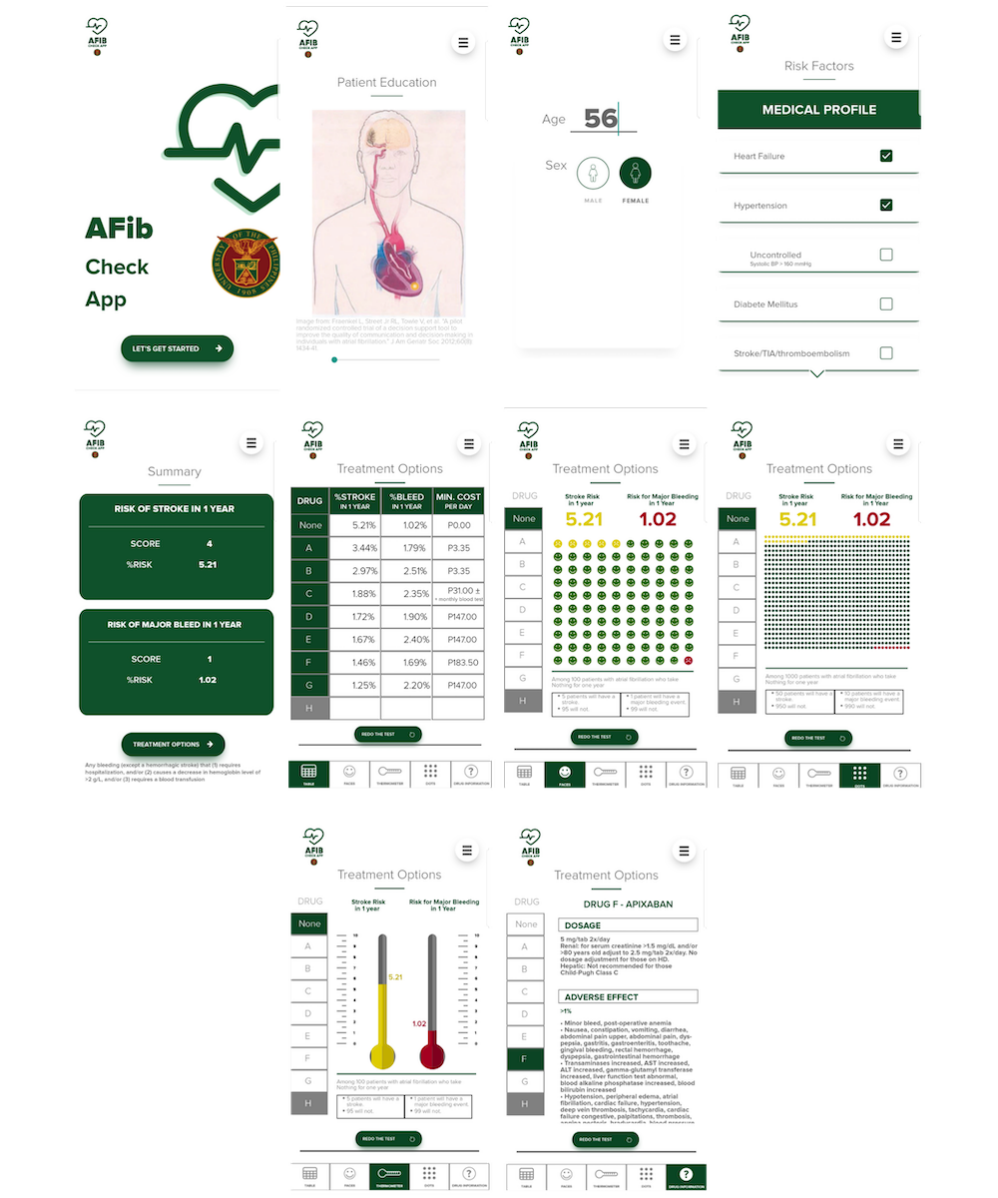
Screenshots of the patient decision aid prototype.

Based on literature review, scoping, and inputs from the focus groups of patients and clinicians, a mobile app was developed as a point-of-care PDA to support the decision-making process of patients with nonvalvular AF regarding their choice of oral anticoagulation. It contains basic information about AF and the different choices for oral anticoagulation among nonvalvular AF for stroke prevention. This can be accessed offline and is intended to be used in the setting of a clinical consultation in order to facilitate communication and discussion of treatment options between patients and clinicians. A modified version of a validated image [23] for patient education was incorporated into the PDA prototype in order to aid clinicians to explain to patients how their condition can predispose them to the development of an ischemic stroke. It also includes a risk calculator that computes for individualized baseline stroke and bleeding risk using mCHA2DS2VASc (modified congestive heart failure, hypertension, age ≥75, diabetes mellitus, stroke or transient ischemic attack, vascular disease, age 50 to 74, sex) and HASBLED (hypertension, abnormal renal and liver function, stroke, bleeding predisposition, labile INRs, elderly, drugs or alcohol) scores. The current locally available oral anticoagulant choices include aspirin, warfarin, apixaban, rivaroxaban, and dabigatran. Individual data of the drugs’ stroke risk reduction and bleeding risk were compared using the data from a 2017 network meta-analysis [20]. The latest market price has also been presented. Medications have been anonymized using letters to facilitate unbiased decision-making based on the factors identified in the early phase of the study. These factors are the drug’s stroke risk reduction, bleeding risk, and cost. Individualized stroke risks and bleeding risks were presented as three pictographs as follows: (1) number of events among 100 people in 1 year, (2) the number of events among 1000 people in 1 year, and (3) a thermometer scale with percentages of stroke risk and major bleeding episodes in 1 year. Once a decision is made, the name of the chosen drug, together with relevant information on dosing and diet advise, can be revealed by tapping on the letter that corresponds to the drug of choice. Screenshots of the app can be seen in Figure 2.

### Pilot Test

We performed pilot testing of the decision aid on a sample of 30 physicians and 37 patients. The demographic characteristics of both patients and physicians are summarized in Tables 1 and 2.

As seen in Table 3, the use of the PDA resulted in a significant decrease in the total DCS score and all its subscale scores. The total DCS score showed a decrease by 35 points (100-point scale) (*P*<.001). Only 8% of the patients had a total DCS score <25 (associated with implementing decisions) before PDA implementation compared with 73% after its implementation. Similarly, 68% of patients had a total DCS score >37.5 (associated with decision delay or feeling unsure about implementation) before PDA implementation compared with 3% after its implementation.

**Table 1:** Patient characteristics.

Characteristic		Value (N=37)
Age (years), mean (SD)		61 (11)
**Sex, n (%)**		
	Male	27 (73%)
	Female	10 (27%)
**Highest educational attainment, n (%)**		
	Elementary	12 (32%)
	High school	13 (35%)
	College	4 (11%)
	Vocational	3 (8%)
	Postgraduate	5 (14%)
**Annual household income (PHP^a^), n (%)**		
	Less than 80,000	35 (94%)
	80,000-160,000	1 (3%)
	320,000-400,000	1 (3%)

^a^PHP: Philippine peso.

**Table 2:** Physician characteristics.

Characteristic		Value (N=30)
Age (years), mean (SD)		29 (2)
**Sex, n (%)**		
	Male	19 (63%)
	Female	11 (37%)
**Specialization, n (%)**		
	Internal medicine	20 (67%)
	Cardiology	8 (27%)
	Neurology	2 (7%)

**Table 3:** Decisional conflict scores (N=37).

Variable	Pre-PDA^a^ score, mean (SD)	Post-PDA score, mean (SD)	*P* value (two-tailed, paired)
Total DCS^b^ score^c^	48.73 (18.49)	13.97 (12.09)	<.001
Uncertainty subscore^d^	43.91 (21.57)	13.73 (15.49)	<.001
Informed subscore^e^	57.20 (22.66)	12.83 (15.66)	<.001
Values clarity subscore^f^	57.88 (45.51)	13.47 (21.17)	<.001
Support subscore^g^	43.46 (20.13)	21.17 (16.26)	<.001

^a^PDA: patient decision aid.

^b^DCS: Decisional Conflict Scale.

^c^Scores range from 0 (no decisional conflict) to 100 (extremely high decisional conflict).

^d^Scores range from 0 (feels extremely certain about the best choice) to 100 (feels extremely uncertain about the best choice).

^e^Scores range from 0 (feels extremely informed) to 100 (feels extremely uninformed).

^f^Scores range from 0 (feels extremely clear about personal values for benefits & risks/side effects) to 100 (feels extremely unclear about personal values).

^g^Scores range from 0 (feels extremely supported in decision-making) to 100 (feels extremely unsupported in decision-making).

Patients also exhibited an increase in the AF knowledge score after the use of the decision aid by 5 points, using a 24-point knowledge tool (mean 10.35, SD 2.43 vs mean 15.4, SD 4.4; *P*<.001). The mean duration of the consults was 15 (SD 6) minutes.

Lastly, we examined the acceptability of the decision aid for both patients and doctors by checking the responses to the Ottawa Acceptability questionnaire (Tables 4 and 5). Acceptability for both patients and doctors was generally high. The percentages of patients who found that the way information was presented was satisfactory were as follows: (1) impact of AF: 98% (36/37), (2) information on risk factors: 95% (35/37), (3) medication options: 98% (36/37), (4) treatment benefits: 98% (36/37), and (5) treatment risks: 95% (35/37). In addition, 92% (34/37) of patients found the length of material to be just right, 92% (34/37) found the calculated values easy to understand, all patients found the amount of information to be just right, and all agreed that the material was balanced, helpful, and with enough information to decide.

**Table 4:** Patient acceptability (presentation of information) (N=37).

Presentation of information	Value, n (%)^a^			
	Poor	Fair	Good	Excellent
Impact of atrial fibrillation	0 (0%)	1 (3%)	11 (30%)	25 (68%)
Risk factors	0 (0%)	2 (5%)	10 (27%)	25 (68%)
Medication options	0 (0%)	1 (3%)	9 (24%)	27 (73%)
Treatment benefits	0 (0%)	1 (3%)	9 (24%)	27 (73%)
Treatment risks	0 (0%)	2 (5%)	11 (30%)	24 (65%)

^a^The percentages do not add up to 100% because of rounding error.

**Table 5:** Patient acceptability (other measures) (N=37).

Other measures		Value, n (%)
**Length of material**		
	Too long	2 (5%)
	Too short	1 (3%)
	Just right	34 (92%)
**Amount of information**		
	Too much	0 (0%)
	Too little	0 (0%)
	Just right	37 (100%)
**Balanced presentation**		
	Slanted	0 (0%)
	Balanced	37 (100%)
**Use in decision-making**		
	Useful	37 (100%)
	Not useful	0 (0%)
**Understandability**		
	Easy	34 (92%)
	Difficult	3 (8%)
**Information e** **nough to** **d** **ecide**		
	Yes	37 (100%)
	No	0 (0%)

Thirty doctors pilot tested the mobile app. They found that the decision aid was easy to use (87%), easy to understand (90%), and easy to experiment with (80%). Moreover, they agreed that this strategy is reliable (90%), is better than their usual way of helping patients decide (90%), is compatible with how they think things should be done (97%), is cost-effective compared with their usual approach (80%), will save them time (67%), has results that are easy to see (90%), and will result in patients making more informed decisions (93%). Moreover, most (90%) doctors thought that the components of the aid can be used by themselves. The majority (90%) of the doctors who used the aid found that it complements their usual approach, which means that they do not need to make major changes to the way they do things (73%). Lastly, most of them thought that the use of the aid will cause more benefit than harm (93%) and that it is suitable for helping patients make value-laden choices (94%).

## Discussion

### Principal Findings

In this paper, we describe the development process of a decision aid to help patients with nonvalvular AF choose among the available options for anticoagulation for stroke prevention. Our PDA was effective in reducing patient decisional conflict and increasing patient knowledge, and was acceptable to doctors and patients alike. It is meant to facilitate a two-way communication between the doctor and patient, thus involving both parties in decision-making. Currently, this is the only PDA developed for patients with nonvalvular AF in our setting.

Our PDA development was guided by the IPDAS systematic process to ensure that it adheres to international standards [8]. Since the population in which we tested our decision aid mostly included patients with a low socioeconomic status, who were also more likely to have a lower health literacy [24], special attention was paid to make the PDA easily comprehensible. In addition, our PDA is meant to be downloaded to the clinician’s device and used in the clinic during consultation as an adjunct tool to facilitate communication and discussion of the different treatment options. As suggested by current available evidence [24], successful health literacy interventions should be delivered by a health professional and must be designed using plain language, simple numbers, and visual techniques. These features were incorporated in the design of our decision aid.

Our pilot study demonstrated that this decision aid was generally acceptable in the Philippine setting. In the FGDs, both doctors and patients expressed interest in participating in SDM when presented with the opportunity. This was also reflected by the predominantly positive responses when we assessed for the acceptability of the PDA on pilot testing. This finding is of great importance to the evidence base for SDM and decision aids in general since most studies on the subject are conducted in Western countries. This finding is also consistent with the finding of a previously published study on the use of a decision aid in the Filipino population [17].

The noted reduction in patient decisional conflict is particularly significant since it provides preliminary evidence regarding the effectiveness of our decision aid as a decision support intervention, as previously documented by a systematic review [10]. Participants showed a change from a score that is associated with decision delay and uncertainty prior to the use of the decision aid to a score that is associated with implementing decisions after its use. This is indicative of effective decision-making where patients are likely to make an informed choice that is consistent with their personal values [25]. Often, it is seen to be translatable to more patient satisfaction and adherence to the choice made. In contrast, a higher decisional conflict is seen as an independent predictor of blame for bad outcomes [26,27]. It has been found that for every unit increase in the DCS score, patients are 19% more likely to blame their doctor for bad outcomes [26].

Several limitations must be acknowledged. As this was a pilot study that used a before-and-after study design, our findings and analyses are derived from a relatively small convenience sample of resident doctors and patients from a single center, which may have generalizability issues. Moreover, we only explored the effectiveness of the decision aid in terms of immediately observable outcomes, that is, decisional conflict, knowledge, and applicability. A randomized controlled trial, therefore, is warranted to explore other outcomes, such as choice adherence, in a larger sample while comparing the use of PDAs to usual care.

### Conclusions

The developed app-based PDA, which was designed to help patients with nonvalvular AF choose among different anticoagulation options for stroke prevention, (1) reduced patient decisional conflict, (2) improved patient knowledge, and (3) was acceptable to patients and physicians in the local setting. Future research should focus on further testing its effectiveness compared to usual care using a randomized controlled trial. Decision aids for other conditions should also be developed.

## Abbreviations

AF: atrial fibrillation
DCS: Decisional Conflict Scale
FGD: focus group discussion
IPDAS: International Patient Decision Aid Standards
PDA: patient decision aid
SDM: shared decision-making
UP-PGH: University of the Philippines – Philippine General Hospital
